# Therapeutic Potential of Remote Ischemic Conditioning in Vascular Cognitive Impairment

**DOI:** 10.3389/fncel.2021.706759

**Published:** 2021-08-03

**Authors:** Rui Xu, Qianyan He, Yan Wang, Yi Yang, Zhen-Ni Guo

**Affiliations:** ^1^Department of Neurology, Stroke Center & Clinical Trial and Research Center for Stroke, The First Hospital of Jilin University, Changchun, China; ^2^China National Comprehensive Stroke Center, Changchun, China; ^3^Jilin Provincial Key Laboratory of Cerebrovascular Disease, Changchun, China

**Keywords:** vascular cognitive impairment, remote ischemic conditioning, cerebral blood flow, white matter, neurovascular unit, inflammation, oxidative stress

## Abstract

Vascular cognitive impairment (VCI) is a heterogeneous disease caused by a variety of cerebrovascular diseases. Patients with VCI often present with slower cognitive processing speed and poor executive function, which affects their independence in daily life, thus increasing social burden. Remote ischemic conditioning (RIC) is a non-invasive and efficient intervention that triggers endogenous protective mechanisms to generate neuroprotection. Over the past decades, evidence from basic and clinical research has shown that RIC is promising for the treatment of VCI. To further our understanding of RIC and improve the management of VCI, we summarize the evidence on the therapeutic potential of RIC in relation to the risk factors and pathobiologies of VCI, including reducing the risk of recurrent stroke, decreasing high blood pressure, improving cerebral blood flow, restoring white matter integrity, protecting the neurovascular unit, attenuating oxidative stress, and inhibiting the inflammatory response.

## Introduction

Vascular cognitive impairment (VCI) is the second most common type of cognitive dysfunction following Alzheimer’s disease (AD) among those aged 65 years or older (Rockwood et al., 2000). In developing countries such as China, vascular-related mild cognitive impairment (MCI) is the most common type, accounting for 42% of all MCI cases (Jia et al., 2014). Impairments in the processing speed and executive function are distinctive symptoms of VCI, which significantly interfere with the ability to maintain independent living and increase the burden on society (Sachdev et al., 2004; Wallin et al., 2018). With an increasingly aging population, VCI is causing an increasing social burden. To this end, we should explore more effective treatment methods to control VCI.

Currently, interventions for VCI are largely based on prevention. Pharmacotherapy for VCI shows mild improvement and is majorly limited to vascular dementia (VaD), the severe form of VCI (Gorelick et al., 2011), or VCI mixed with AD (Shaji et al., 2018). VCI involves multiple pathobiological changes. Cerebral hypoperfusion and damage to the neurovascular unit (NVU) are postulated as the main pathobiological mechanisms in VCI (Sinha et al., 2020; Song et al., 2020). In addition, oxidative stress and the inflammatory response have also been identified as important contributors. Therefore, a “multitarget” therapeutic intervention may prove to be more effective in managing this disease.

Remote ischemic conditioning (RIC) is a non-pharmacological and non-invasive intervention that can activate multiple biological signaling pathways to generate endogenous neuroprotective effects (Hao et al., 2020; Ripley et al., 2021). Recently, RIC has emerged as a promising intervention for the prevention and treatment of cognitive impairment due to cerebrovascular disorders (see Table 1). A better understanding of the mechanisms in this process will assist clinicians in translating this simple and efficient therapy into clinical benefits.

**TABLE 1:** Clinical and animal evidence of RIC in VCI.

Clinical study							

Patients	Location of RIC	Timing of RIC	Cycles	Protocol	Cognitive function	Physiological/molecular mechanisms	References
Patients with first-time non-cardiac ischemic stroke (*n* = 104)	Upper limb	Within 14 days after an ischemic stroke	5 min × 5 min	Daily for 6 months	↑MoCA	↑Cerebral hemodynamics ↓ICAM-1,ET-1	Feng et al., 2019
Patients with post-stroke cognitive impairment (*n* = 48)	Upper limb	Not mentioned	4 min × 5 min	Daily for 7 days	↑MoCA ↑ADAS-cog	Not mentioned	Li et al., 2020
Patients with subcortical ischemic vascular dementia (*n* = 37)	Bilateral upper limb	Cognitive impairment lasting for at least 3 months	5 min × 5 min	Daily for 6 months	↑JLO	↓WMLV ↓Hs-CRP	Liao et al., 2019
Patients with intracranial arterial stenosis (*n* = 58)	Bilateral upper arm	Within 7 days after an ischemic stroke or TIA	5 min × 5 min	Twice daily for 300 days	↑MoCA,MMSE	↓WMHs	Zhou et al., 2019
Patients with cerebral small-vessel disease-related mild cognitive impairment (*n* = 30)	Bilateral upper limb	Cognitive impairment lasting for at least 3 months	5 min × 5 min	Twice daily for 1 year	↑Visuospatial and executive ability	↓WMHs ↓Pulsation indices in MCA ↓Plasma triglyceride, total cholesterol, low-density lipoprotein, and homocysteine	Wang Y. et al., 2017
Patients with cerebral small vessel disease (*n* = 17)	Bilateral upper arm	Not mentioned	5 min × 5 min	Twice daily for 1 year	No effect	↓WMHs ↑Flow velocity in MCA	Mi et al., 2016

**Animal study**							

**Animal model**	**Location of RIC**	**Timing of RIC**	**Cycles**	**Protocol**	**Cognitive function**	**Physiological/molecular mechanisms**	**References**

SD rats; BCAO	Bilateral hind limbs	Three days after the hypoperfusion	3 min × 10 min	Daily for 28 days	↑Morris water maze	↑CBF, angiogenesis ↑NOS/NO	Ren et al., 2018a
C57BL/6J mice; BCAS	Bilateral hind limbs	One week after the surgery	4 min × 5 min	Daily for 1 month or 4 months	↑NOR test, Y-maze test	↑WM integrity ↑CBF, angiogenesis	Khan et al., 2018
C57BL/6J mice; BCAS	Bilateral hind limbs	One week after the surgery	4 min × 5 min	Daily for 2 weeks	↑NOR test	↑WM integrity	Khan et al., 2015
						↓neuroinflammation	
						↓Aβ aggregation	
SD rats; BCAO	Bilateral hind limbs	Three days after the hypoperfusion	3 min × 10 min	Daily for 28 days	↑Morris water maze	↑WM integrity ↑PTEN/Akt/mTOR	Li et al., 2017
Wistar rats; occluding both carotid arteries for 20 min, followed by reperfusion	Bilateral hind limbs	After the surgery	3 min × 10 min	Once	↑Morris water maze, passive avoidance task, Y-maze	↓Oxidative stress, inflammation ↑HO-1,BDNF	Ramagiri and Taliyan, 2017b
Wistar rats; occluding both carotid arteries for 20 min, followed by reperfusion	Bilateral hind limbs	Immediately following reperfusion	3 min × 10 min	Once	↑Morris water maze, passive avoidance task, Y-maze	↓Oxidative stress, inflammation ↑GSK-3β/CREB/BDNF	Ramagiri and Taliyan, 2017a
Wistar mice; BCAS	Bilateral hind limbs	Two weeks after surgery	4 min × 5 min	Daily for 2 weeks	↑ NOR test	↑Autophage	Wang H. et al., 2017
Swiss albino mice; occluding both carotid arteries for 20 min, followed by reperfusion	Hind limb	Before surgery	4 min × 5 min	Once	↑Morris water maze	↓Oxidative stress	He et al., 2019
SD rats; tMCAO for 60 min	Right hind-limb	Before surgery	3 min × 5 min	Once	↑Morris water maze	↑Cholinergic neurons in CA1 region	Hu et al., 2013
SD rats; occluding both carotid arteries for 20 min, followed by reperfusion	Right hind-limb	Before surgery	3 min × 10 min	Once	↑Morris water maze	↑Bcl-2	Xu et al., 2011
BULB/C mice; occluding both carotid arteries for 20 min, followed byreperfusion	Renal artery	24 h before surgery	3 min × 5 min	Once	↑Passive avoidance test	↑mTOR, SOD	Zare Mehrjerdi et al., 2013

*MoCA, montreal cognitive assessment scale; ADAS-cog, Alzheimer’s disease assessment scale-cognitive; JLO, judgment of line orientation; MMSE, mini-mental state examination; WM, white matter; WMLV, white matter lesion volume; WMHs, white matter hyperintensities; ICAM-1, intercellular adhesion molecule-1; ET-1, endothelin-1; Hs-CRP, high-sensitivity C-reactive protein; MCA, middle cerebral artery; NOR test, novel object recognition test; eNOS, endothelial nitric oxide synthase; NO, nitric oxide; PTEN, phosphatase and tensin homolog; mTOR, mechanistic target of rapamycin; HO-1, hemeoxygenase -1; BDNF, brain derived neurotrophic factor; GSK-3β, glycogen synthase kinase 3 beta; CREB, cAMP response element-binding protein; CA1, cornu ammonis; Bcl-2, B-cell lymphoma 2; SOD, superoxide dismutase.*

In this review, we focus on the evidence of the protective effects of RIC on VCI from basic and clinical research. It includes the modifiable effect of RIC on the risk factors and pathobiologies of VCI, such as recurrent stroke, hypertension, cerebral blood flow (CBF), white matter (WM) injury, NVU, oxidative stress, and inflammation response.

## Vascular Cognitive Impairment

Vascular cognitive impairment refers to a decline in cognitive function attributable to vascular risk factors or cerebrovascular diseases as well as mixed pathologies such as AD (Gorelick et al., 2011; Skrobot et al., 2017), ranging from mild VCI to major VCI (VaD). Major VCI can be further divided into four subtypes: post-stroke dementia, subcortical ischemic vascular dementia, multi-infarct dementia (MID), and mixed dementia (Skrobot et al., 2017).

### Brief History of VCI

The evolution of the terminology used to characterize cognitive impairment with a vascular origin reflects our improvement in understanding this disease. Approximately 40 years ago, the term MID was introduced to describe cognitive deficits due to large or small cerebral infarctions (Hachinski et al., 1974). It was considered as one of the two main pathologies of dementia, the other being “Alzheimer like changes” (e.g., senile plaques, neurofibrillary tangles, and granulovacuolar degeneration). Later, a variety of vascular pathologies have been shown to be associated with cognitive impairment, and thus the term “VaD” was used for a more accurate generalization of this disease (Roman et al., 1993). However, cognitive decline is a consecutive process, and patients with MCI may be ignored using this term. VCI has been proposed to cover the full spectrum of cognitive impairment. Other terminologies are also widely used, such as vascular cognitive impairment and dementia. Here, we use “VCI” to refer to the full range of severity of this disease.

### Risk Factors of VCI

Over the past decades, a substantial drop in the incidence and prevalence of dementia has been observed in high-income countries (Hachinski et al., 2019). A combination of improved management of vascular risk factors and increased cognitive reserve (such as a higher level of education) may explain the trend (Wu et al., 2017).

Stroke is a strong modifiable risk factor for VCI (Portegies et al., 2016). It has been reported that 40% of stroke patients exhibit MCI, 1 year after stroke onset (Sexton et al., 2019). Moreover, 10% of patients with first-ever stroke develop new-onset dementia, and about 40% of patients with recurrent stroke have dementia (Pendlebury and Rothwell, 2009). Compared with stroke-free individuals, stroke increases the risk of dementia by almost twofold and the risk of recurrent stroke by threefold (Kuzma et al., 2018).

Hypertension is another important modifiable risk factor for VCI. There is consistent evidence that hypertension, especially midlife (45–55 years of age) hypertension is associated with late-life cognitive decline and VCI (Launer et al., 2000; Yamada et al., 2009; Gottesman et al., 2014). For individuals with prehypertension [systolic blood pressure (SBP) of 120–139 mmHg or diastolic blood pressure (DBP) of 80–89 mmHg], the risk of dementia was almost as high as that of patients with hypertension, and approximately 30% higher than that of patients with normotension (Gottesman et al., 2017).

Other modifiable risk factors include hypercholesterolemia, diabetes mellitus, alcohol consumption, obesity, depression, and smoking, where diabetes mellitus is a well-established risk factor for all causes of dementia (Gorelick et al., 2011; Iadecola et al., 2019). As for non-modifiable risk factors, there is a strong link between aging and VCI. Aging is associated with VaD, post-stroke dementia, unspecified dementia, and Alzheimer’s dementia (Yang T. et al., 2017; Iadecola et al., 2019). In contrast, higher education and physical activities are thought to have a protective role in VCI (Najar et al., 2019; Jia et al., 2020).

### Pathobiologies of VCI

The underlying pathobiologies of VCI have been attributed to multiple mechanisms. Altered vascular structures or functions, such as cerebral hypoperfusion, blood brain barrier (BBB) disruption and CBF dysregulation, have long been implicated in the pathogenesis of VCI (Iadecola, 2013). Disruption of the structural and functional integrity of the NVU is increasingly recognized as a key contributor to VCI, which provides a conceptual framework for studying the pathogenesis of VCI (Li et al., 2021). Additionally, other mechanisms have also been suggested to be involved in this process, including but not limited to oxidative stress, neuroinflammation, and impairment of glymphatic system clearance (Joutel and Chabriat, 2017; Zlokovic et al., 2020). It seems that these mechanisms interact with each other, and it is difficult to identify a temporal and causal relationship between them.

#### Cerebral Hypoperfusion and CBF Dysregulation

There is a general consensus that cerebral hypoperfusion is an etiological factor for VCI. Upon an acute or chronic reduction in CBF, the oxygen and nutrient supply to the brain is compromised, leading to tissue damages both in the gray matter and/or WM (van der Flier et al., 2018). These pathological changes can affect brain network integrity and impair cognitive function (Cremers et al., 2020). The causal relationship between cerebral hypoperfusion and VCI is supported by both human and animal studies (Duncombe et al., 2017; Wolters et al., 2017; Moonen et al., 2021). However, there are contrary observations of decreased CBF being a result of WMHs or aging of the brain instead of a casual mechanism of VCI pathology (van der Veen et al., 2015; Shi et al., 2016), especially when accounting for genetic diseases, such as cerebral autosomal dominant arteriopathy with subcortical infarcts and leukoencephalopathy, where WMHs may appear before the decline of CBF (Joutel and Chabriat, 2017). One possible explanation for this may be the disruption of the NVU (see section “Disruption of the Neurovascular Unit”). Thus, the casual role of cerebral hypoperfusion in VCI remains inconclusive and should be confirmed in future studies.

With a high metabolic rate but low energy storage, the brain needs a rapid and constant supply of CBF to maintain normal function. Cerebral autoregulation (CA) is one of the intrinsic mechanisms of regulating global CBF, and another mechanism of regional CBF regulation is neurovascular coupling (NVC) (see next part). CA enables the brain to maintain a stable CBF while BP fluctuates within a certain range, normally between and 60–150 mmHg (Lassen, 1964). When CA is impaired, cerebral perfusion passively follows BP fluctuation, which may lead to critical consequences in the microcirculation (Shekhar et al., 2017) and to cognitive impairment. It has been demonstrated that CA is often disturbed in patients with cerebrovascular diseases and is strongly associated with WMHs in the elderly (Immink et al., 2005; Purkayastha et al., 2014). However, it is unclear whether impaired CA is a cause or a consequence of VCI.

#### Disruption of the Neurovascular Unit

The NVU is an interconnected functional and anatomical structure composed of neurons, interneurons, glia, vascular cells, and the extracellular matrix (Levit et al., 2020). Components within the NVU act in concert to meet the energy demands of neuronal activity through the regulation of CBF. Meanwhile, they also play an important role in maintaining BBB integrity, trophic support, and waste clearance (Iadecola, 2017). It is not surprising that any damage to these cells will lead to dysfunction of the NVU, compromising cerebral hemodynamics and homeostasis, which in turn affect cognitive function. For instance, hypertension, a major risk factor of VCI, has a detrimental effect on the cerebrovascular system, from the large arteries to the capillaries, leading to cerebral hypoperfusion and NVU damage (de Montgolfier et al., 2019b; Presa et al., 2020). Moreover, elevated intravascular pressure can also directly cause BBB leakage and NVU disruption by targeting endothelial cells (Arlier et al., 2015), activating the glia (Eldahshan et al., 2019), and increasing ROS production (Faraco et al., 2016).

Neurovascular coupling is one of the most studied functions of the NVU, which emphasizes the active regulation of regional CBF in response to neural activity. The cellular and molecular bases of NVC have been previously well elucidated (Iadecola, 2017; Hosford and Gourine, 2019). In brief, neurons act as an initiator in the vascular response, which activates the downstream cells (interneurons, astrocytes, and endothelial cells), and finally leads to relaxation of vascular smooth muscle cells (VSMCs) or pericytes at different vascular levels (arteries or capillaries). This finely tuned regulation of CBF provides sufficient oxygen and nutrients for synaptic activities. Disruption of NVC will lead to cerebral hypoperfusion and contributes to the development of VCI. Neurovascular uncoupling has been demonstrated in various VCI animal models, such as of aging, hypertension, and cerebral hypoperfusion, indicating the important role of NVC in the pathogenesis of VCI (de Montgolfier et al., 2019a; Tarantini et al., 2019b; Chandran et al., 2020).

Pericytes have increasingly become a focus in VCI research. They comprise a pluripotent mural cell type mainly located in the capillaries, precapillary arterioles, and postcapillary venules (Uemura et al., 2020). Recently, a post-mortem study showed that there is substantial loss of pericytes in the WM of patients with VCI compared with controls without dementia (Ding R. et al., 2020). Several studies have reported that chronic or acute loss of pericytes can cause neurovascular uncoupling, followed by WM damage and cognitive impairment, indicating the important role of pericytes in CBF regulation (Montagne et al., 2018; Kisler et al., 2020). However, it remains unclear whether pericytes affect NVC directly via their contractility or indirectly through signal transmission. In contrast, there is evidence against the involvement of pericytes in the regulation of CBF (Hill et al., 2015). Moreover, pericyte loss was reported to cause BBB leakage via endothelial transcytosis in a rat model of CCH, which preceded neuroinflammation and WM damage (Sun et al., 2021). Furthermore, dysfunction of pericytes may also instigate BBB disruption through the increased secretion of collagen *in vitro* (Ozkan et al., 2021). Taken together, loss or dysfunction of pericytes can result in neurovascular uncoupling, BBB disruption, and cerebral hypoperfusion, which, in turn, lead to WM damage and cognitive impairment.

Endothelial dysfunction is a common feature of VCI caused by either large or small cerebrovascular diseases, which is tightly linked to CBF dysregulation, neuroinflammation, NVU dysfunction and loss of trophic support to glial cells (Pi et al., 2018). Abnormalities in NO production and endothelial nitric oxide synthase (eNOS) activity are important markers of endothelial dysfunction (Vanhoutte et al., 2016). Reduced NO bioavailability leads to vasoconstriction and compromised the regulation of CBF, which may exacerbate cerebral hypoperfusion and cognitive decline (Matin et al., 2018; Tarantini et al., 2018, 2019b). In support of this perspective, previous autopsy studies have reported a reduced endothelial-mediated vasodilation in WM lesions in VCI patients (Bagi et al., 2018). Furthermore, in the setting of inflammation, activated ECs can upregulate the expression of intercellular adhesion molecule-1 (ICAM-1) and vascular cell adhesion molecule-1 (VCAM-1). These molecules promote the adhesion and migration of neutrophils and monocytes through the vascular wall and evoke neuroinflammation by stimulating glial cells. Activated endothelial cells have also been proved to increase vascular permeability by opening the intercellular junctions and may allow toxic proteins to leak into the central nerve system (CNS) (Donato et al., 2018). Moreover, ECs play a vital role in supporting the oligodendrocyte lineage cells within the oligovascular niche (Ohtomo and Arai, 2020). For instance, hypoxia-ischemia induces oligogenesis around vessels in a short duration, while it was inhibited with an injury of ECs occurring at the early stage (Wang X. et al., 2020). Thus, endothelium is a crucial component within the NVU, which provides a therapeutic target in the management of VCI.

#### Oxidative Stress

Oxidative stress refers to the imbalance between pro-oxidant and antioxidant systems, which results in the excessive production of reactive oxygen species (ROS) (Wojsiat et al., 2018). Patients with VCI have been reported to have higher levels of malondialdehyde (MDA) (Gustaw-Rothenberg et al., 2010), the end product and the hallmark of lipid peroxidation, and an altered antioxidant defense system, such as decreased superoxide dismutase and glutathione reductase (GR) (Casado et al., 2008). Nicotinamide adenine dinucleotide phosphate oxidases (NOXs) and the mitochondrial respiratory chain are the main sources of ROS (Choi et al., 2014; He et al., 2020). In VCI animal models, CCH and cerebral ischemia/reperfusion (I/R) injury lead to mitochondrial dysfunction and increased NOX expression, followed by an overload of ROS, lipid peroxidation, DNA fragmentation, protein oxidation, and phosphorylation. Finally, these pathological changes cause BBB disruption, endothelial dysfunction, neuronal damage, and cognitive decline (Choi and Lee, 2017; Han et al., 2020). As a matter of fact, oxidative stress is not only associated with VCI, but also plays a detrimental role in the pathogenesis of the risk factors for VCI, which contribute to the progression of cognitive impairment (Liyanagamage and Martinus, 2020; Touyz et al., 2020). Thus, targeting oxidative stress represents a potential therapy for the prevention and management of VCI.

#### Inflammation

Neuroinflammation, which is defined as inflammation involved in the CNS, is a common feature of VCI models. In bilateral common carotid artery occlusion (BCCAO) models, endothelial activation is likely to be an early event under chronic hypoperfusion. Activated ECs facilitate the attachment and extravasation of leukocytes across the BBB. These leukocytes, inflammatory cytokines, and blood products initiate the neuroinflammation by activating glia cells, which in turn aggravate WM damage and BBB injury (Duncombe et al., 2017). Furthermore, neuroinflammation can be enhanced by elevated systemic inflammation through the disrupted BBB. Aging, as the major risk factor for VCI, induces a shift in pro-inflammatory gene expression in ECs and VSMCs in the peripheral, including the induction of inflammatory cytokines, chemokines and adhesion molecules. This pro-inflammatory environment activates and recruits immune cells and ultimately induces a systemic inflammatory response. The resulting vascular inflammation leads to vascular dysfunction, BBB disruption, and consequentially exacerbated the development of VCI (Ungvari et al., 2018).

## Remote Ischemic Conditioning

Remote ischemic conditioning is a safe, simple, and cost-efficient approach to alleviate tissue damage in remote organs, such as the brain, the heart, and the kidney. By pressuring the limb repeatedly at a distance from the target organ using a blood pressure cuff, RIC can induce a non-lethal ischemic condition, which in turn stimulates the body and triggers endogenous protective mechanisms (Hess et al., 2015).

RIC was initially used as an once-occasion treatment to mitigate ischemic/reperfusion injury. Later, it was found that RIC has additional pleiotropic effects, such as improved endothelial function, anti-inflammation, and inhibition of platelet aggregation (Hausenloy et al., 2016). However, the protection period of single RIC is relatively short [generally for 72 h (Ren et al., 2008)], which may prevent its application in chronic conditions. To this end, repetitive daily RIC, termed chronic RIC, has gained much attention. It is able to confer a continuous and long-lasting effect, and thus, is more beneficial for the treatment of chronic diseases (Ding J. Y. et al., 2020). Although many studies on RIC have been carried out, the underlying mechanism still needs to be elucidated. Interestingly, it has been proposed that chronic RIC shares many common mechanisms with physical activities and is considered equivalent to exercise. The latter is currently recognized as an effective treatment for cognitive impairment (Zhao et al., 2018; Geng et al., 2021).

## Role of RIC in Mitigating VCI

Evidence has been shown that RIC can modify VCI-related risk factors and decrease its pathobiological alterations, which protect against cognitive decline and even improve cognitive function.

### RIC Modifies the Risk Factors of VCI

**FIGURE 1:**
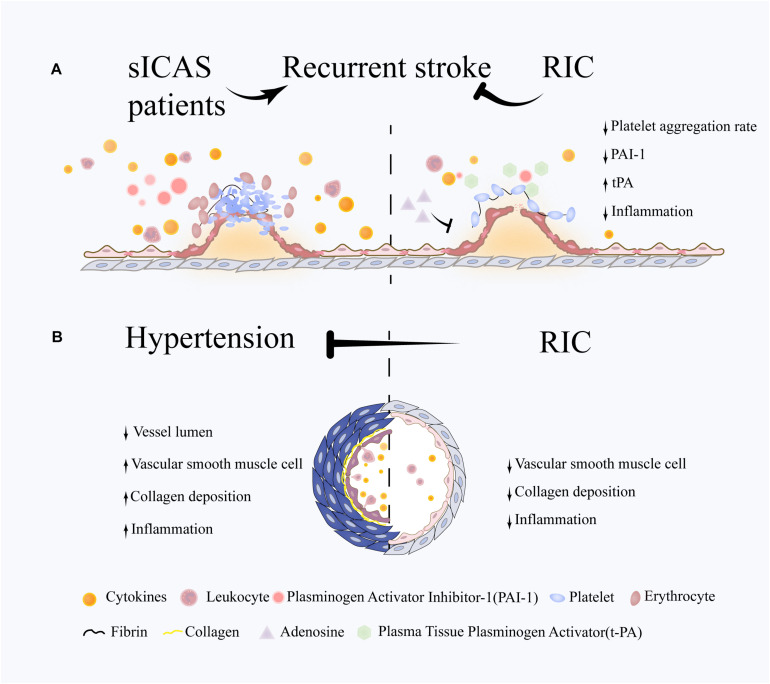
Potential role of RIC on VCI-related risk factors. **(A)** Following an abrupt rupture of an atherosclerotic plaque, platelets respond rapidly through adhesion and aggregation, then thrombins generate a fibrin network to reinforce platelet aggregation and trap a large number of red blood cells, leading to the formation of thrombosis and vascular occlusion. The immune system are also involved in this process through activating the leukocytes and releasing large amount of cytokines. RIC is proven to reduce stroke recurrence in sICAS patients. The underlying mechanisms may involve the attenuation of excessive platelet aggregation and inflammation and the promotion of coagulation system. **(B)** Hypertension is characterized by and increased vascular resistance, which plays an important role in VCI. Vascular remodeling occurs at the early stage in hypertension and is associated with chronic inflammation response, hypertrophy of vascular smooth muscle cells (VSMCs) and deposition of extracellular matrix. RIC is efficient in blood pressure lowering in both hypertensive and prehypertensive patients. One of underlying mechanisms is the suppression of pathological vascular remodeling and downregulation of inflammation response.

Several risk factors that contribute to vascular injury and brain network disruption are modifiable. RIC may prevent VCI by reducing recurrent stroke and controlling high blood pressure (see Figure 1).

Patients with symptomatic intracranial atherosclerosis (sICAS), referred to as luminal stenosis >50%, are at a high risk of recurrent stroke (Hurford et al., 2020), even with standard single antiplatelet therapy (Chimowitz et al., 2005; Mazighi et al., 2006; Liu et al., 2015). Recently, the THALES trial showed that the use of dual antiplatelet therapy (DAPT) for 30 days may lead to a further reduction in the risk of early recurrence in patients with large artery atherosclerosis (Johnston et al., 2020). However, the long-term effects and safety of DAPT remain unknown.

#### Reduced Recurrent Stroke

Remote ischemic conditioning is a safe treatment and may be used as an adjuvant approach to the current standard therapy to reduce recurrent stroke in patients with sICAS. In a randomized RIC study, 68 sICAS patients (<80 years) who had a stroke or transient ischemic attack (TIA) within 30 days were consecutively enrolled, and all patients received standard medical treatment (Meng et al., 2012). RIC was performed using an electric device with blood pressure cuffs inflated to 200 mmHg for 5 min and deflated for 5 min for five cycles on bilateral arms twice daily. The rate of stroke recurrence in the RIC group was 5 and 7.9% at 90 and 300 days, respectively, compared to 23.3 and 26.7% in the control group. An improvement in cerebral hemodynamics was also observed at the 300 days follow-up. Later, the same study group demonstrated the efficiency of this protocol in patients with sICAS above 80 years of age (Meng et al., 2015). Compared with the control group, RIC showed no difference in terms of safety at the 30 days follow-up, such as local skin integrity and cerebral hemorrhage, while it significantly reduced the incidence of cerebrovascular events (2 infarctions and 7 TIA vs. 8 infarctions and 11 TIA, respectively) after 180 days of treatment. Additionally, the plasma levels of platelet aggregation rate and plasminogen activator inhibitor-1 were downregulated, while plasma tissue plasminogen activator were elevated in the RIC group for the initial 30 days, together with a reduction in hsCRP, interleukin-6 (IL-6), and leukocyte count. The inhibitory effect of RIC on coagulation has also been observed in other clinical and experimental studies (Ropcke et al., 2012; Lanza et al., 2016; Gorog et al., 2021). It was speculated that adenosine may play an important role in this process (Przyklenk and Whittaker, 2017). During the ischemic period of RIC, the concentration of adenosine is elevated, which is a potent inhibitor of platelet function by binding to the adenosine A2 receptor on the platelet surface. Thus, the beneficial effect of RIC on reducing recurrent stroke may be partially attributed to the inhibition of platelet activity.

#### Decreased Blood Pressure

A self-experimentation study on a 72-year-old man with normotension/prehypertension revealed that repeated RIC (twice daily for 10 days) decreased BP and had a persisting effect even 5 days after cessation with no rebound (Madias, 2015). Subsequently, two small self-controlled studies were designed to verify the BP-lowering effect of RIC for a longer period in hypertension/prehypertension patients. The first clinical trial included patients who were newly diagnosed with essential hypertension and did not undergo any medical treatment. The patients received three cycles of RIC on the upper arm for 5 min per cycle for 30 days (Tong et al., 2019). Chronic RIC significantly reduced SBP by 8 mmHg and DBP by 6 mmHg and improved microvascular endothelial function, as measured by the finger reactive hyperemia index. The second study was conducted in elderly patients with prehypertension or grade one hypertension (Gao et al., 2021). None of the patients received any antihypertensive agents. Chronic RIC consists of five cycles of ischemia and reperfusion interval for 5 min per cycle on bilateral upper arms twice daily for 4 weeks. Compared with the baseline, the SBP and DBP were decreased by 10.2 and 5.4 mmHg, respectively, and the arterial stiffness is also improved, as measured by pulse wave velocity. Furthermore, the authors performed RIC in spontaneously hypertensive rats (SHRs), which are the most commonly used genetic animal models to study hypertension and can exhibit cognitive deficits and WMHs similar to those in humans (Lerman et al., 2019). Hypertension results in occlusive vascular remodeling and promotes arterial stiffness in the large cerebral arteries, as well as penetrating arterioles. These pathological changes lead to reduced vessel lumen and compromised cerebrovascular function (Iadecola and Gottesman, 2019). RIC for 6 weeks significantly decreased the BP, reduced the inflammatory response, and suppressed excessive vascular remodeling in the conduit arteries and small resistance arteries of the brain.

Thus, RIC is effective in lowering BP in individuals with hypertension/prehypertension, which may alter cerebral vasculature, improve vascular function, and protect against cognitive decline. Given that most patients with hypertension will receive antihypertensive medication, further investigation is needed to determine whether chronic RIC has synergistic effects with antihypertensive drugs.

Evidence of RIC on other VCI-related risk factors is not currently available. However, one clinical study explored the effect of RIC on vascular and neuronal function in patients with peripheral arterial diseases and type 2 diabetes mellitus (Hansen et al., 2019). The authors reported negative results and found no improvement in tissue oxygenation or other outcomes, indicating that diabetes may have implications for RIC. In contrast, a study has shown that RIC attenuated cerebral I/R injury, even in the presence of diabetes in a transient middle cerebral artery occlusion (tMCAO) animal model (Liu et al., 2020). The underlying mechanisms may be the suppression of excessive inflammation and activation of the brain ERK signaling pathways. However, it is difficult to draw a conclusion based on these limited and inconsistent evidence, but this indicates that diabetes may need to be considered in future studies.

In summary, chronic RIC is efficient in the management of risk factors for VCI. It can be implemented synergistically with antiplatelet therapy to reduce recurrent stroke in patients with sICAS and as an alternative intervention to treat hypertension partially by regulating the pathological process to the vasculature.

### Potential Effects of RIC on VCI Pathobiologies

**FIGURE 2:**
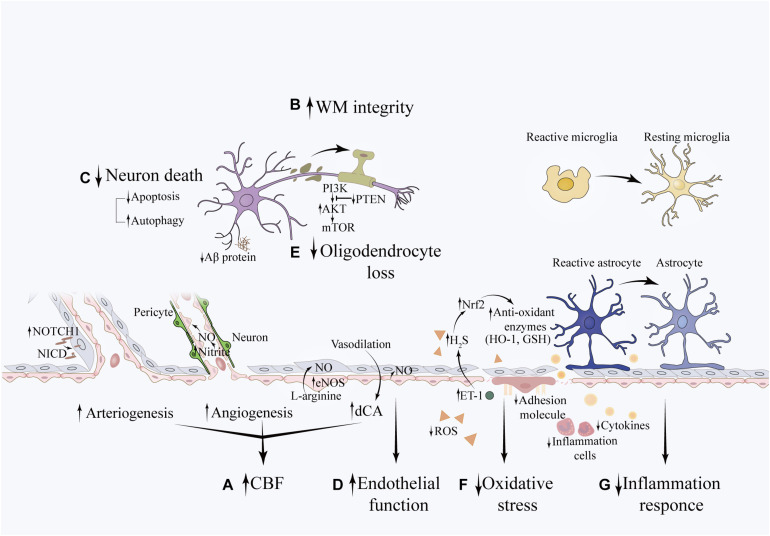
Potential effects of RIC on VCI pathobiologies. The beneficial effects of RIC on VCI is associated with **(A)** increased CFB: the NOTCH1 signaling pathway may be involved in angiogenesis, which increases vascular diameter and promotes collateral formation. The eNOS/Nitrite/NO system plays an important role in the process of angiogenesis. The sheer stress induced by RIC upregulates the synthesis of eNOS in triggered organ and increases the concentration of nitrite in the circulation (not shown in this illustration). Nitrite reduces to NO under hypoxia condition and promotes new capillary formation. In addition, NO is an essential vasoactive agent in the regulation of vascular tension and cerebral autoregulation. **(B)** improved WM integrity **(C)** decreased neuronal death **(D)** restoration of endothelial function **(E)** reduced oligodendrocyte loss: the potential underlying mechanism is associated with decreased apoptosis of oligodendrocytes through activation of the PTEN/Akt/mTOR pathway. **(F)** attenuated oxidative stress: RIC can upregulate the expression of Nrf2 and attenuate the oxidative stress induced by hypoperfusion through promoting the expression of anti-oxidant enzymes, such as GSH and HO-1. This may partially mediated by the elevation of ET-1 in the plasma, which further upregulates the concentration of H_2_S within the brain. **(G)** decreased inflammation response: RIC can decrease the systemic inflammation by downregulating the level of inflammatory cytokines and leukocyte count. Meanwhile, RIC can mitigate neuroinflammation as evidenced by decreased expression of IBA-1 and GFAP, which are the markers of reactive microglial and reactive astrocytes, respectively. The expression of ICAM-1 and VCAM-1, markers of endothelial activation, are also downregulated. In addition, this was accompanied with less deposition of Ab protein.

Pathobiological changes in VCI exhibit reciprocal causation, which aggravates the progression of this disease. Risk factors, such as hypertension and aging have been linked to inflammation, especially chronic inflammation. It promotes oxidative stress and leads to NVU disruption and vascular injury, resulting in compromised CBF and WM integrity, which influences higher-level cognitive process. The protective effects of RIC on VCI occur at different biological levels, encompassing a series of molecular changes (see Figure 2).

#### RIC Improves the Cerebral Blood Flow

Increasing evidence indicates that RIC can improve CBF in acute artery occlusion or chronic cerebral hypoperfusion. It has been reported that a single RIC treatment in preclinical studies significantly enhanced regional CBF after acute ischemic stroke (Hoda et al., 2012, 2014). Meanwhile, repeated daily RIC is efficient in improving global CBF in chronic ischemia conditions, such as sICAS and Moyamoya disease (MMD) (Meng et al., 2012; Ding J. Y. et al., 2020).

The promotion of vascular remodeling, including arteriogenesis and angiogenesis, may underlie the potential mechanisms of RIC on CBF. Arteriogenesis and angiogenesis are critical mechanisms for the maintenance of sufficient cerebral supply after acute or chronic CBF decline and the prevention of consequential cognitive decline (Freitas-Andrade et al., 2020).

Arteriogenesis, also known as collateral formation, leads to dilation and remodeling of preexisting capillaries into larger conductance vessels as a restoration of normal blood flow (Deindl and Quax, 2020). In a tMCAO mouse model, which is the most commonly used animal model of ischemic stroke, repeated daily RIC for 14 days showed a significant increase in CBF and promotion of arteriogenesis, as indicated by an increase in arterial diameter and vascular smooth muscle cell proliferation. And this was associated with elevated expression of Notch1 and Notch intracellular domain in the arteries of the peri-infarct area (Ren et al., 2018b). Notch proteins are a family of transmembrane proteins that can become cleaved and translocate to the nucleus. Activation of Notch1 could promote arteriogenesis in a hindlimb ischemic model, which was suppressed by inhibition or knockdown of NOTCH1 (Kikuchi et al., 2011). This was also confirmed in developmental and other diseases (Tirziu et al., 2012; Cristofaro et al., 2013). Thereby, the Notch signaling pathway plays a key role in regulating arteriogenesis (Limbourg et al., 2007).

Angiogenesis refers to the formation and differentiation of new blood vessels (Cooke and Meng, 2020). It has been reported that RIC facilitates angiogenesis in VCI animal models by virtue of the activation of eNOS/NO/nitrite system (Ren et al., 2018a). NO is an important signaling molecule in the regulation of vascular remodeling, which is mainly produced by ECs via eNOS under ischemic conditions (Jin et al., 2021). Because of its highly reactive nature, NO can be oxidized to nitrite during circulation and reduced to NO in a hypoxic environment (Farah et al., 2018). Thus, nitrite is considered as a “storage” pool of NO and a potential endocrine mediator in the plasma. Several clinical and preclinical studies have consistently reported that the concentration of plasma nitrite is upregulated after RIC treatment (Farah et al., 2018; Ikonomidis et al., 2021). The augmentation of nitrite was dependent on reactive hyperemia during the reperfusion phase of RIC, which leads to an upregulation of eNOS synthesis and NO release in the ECs (Dezfulian et al., 2017). In a VCI mouse model induced by bilateral common carotid artery stenosis (BCAS), chronic RIC was performed as four cycles of altering ischemia and reperfusion to 200 mmHg for 5 min per cycle with a remodeled rodent BP instrument on bilateral hind limbs (56). Compared with the control group, 1 month of RIC significantly increased the plasma nitrite level, enhanced angiogenesis, improved CBF and preserved cognitive function. What is more, the effect of RLIC on angiogenesis maintained for up to 6 months. Consistently, this protective effect of RIC was also confirmed in a rat model of VCI caused by BCCAO (Ren et al., 2018a). In this study, the expression of p-eNOS in the brain was significantly elevated from week 2 and persisted until week 4. Furthermore, intraperitoneal administration of L-NAME, a non-selective NOS inhibitor, abolished the protective effect, indicating an important role of the eNOS/NO/nitrite system in mediating the beneficial effects of RIC.

In addition to vasculature integrity, regulation of CBF is important to ensure proper brain function (Popa-Wagner et al., 2015). In our previous study, we found that a single RIC treatment significantly improved dynamic cerebral autoregulation (dCA), and the effect lasted for at least 24 h after the RIC procedure in healthy individuals (Guo et al., 2019). The effect of chronic RIC on dCA and other CBF regulation mechanisms is worth investigating in patients with VCI.

In conclusion, RIC exerts beneficial effects on CBF by facilitating cerebrovascular angioarchitecture and improving the autoregulation of CBF, which may in turn enhance cognitive function.

#### RIC Promote the White Matter Integrity

Several clinical trials have confirmed that chronic RIC is a promising intervention for WMHs and cognitive decline. In a randomized trial of RIC, 58 patients above 80 years of age with ICAS were randomly assigned to the RIC or sham RIC group (Zhou et al., 2019). Patients in the RIC group received five cycles of ischemia and reperfusion for 5 min per cycle on bilateral upper arms twice daily for 300 days. The severity of WMHs was measured using the Fazekas scale and the Scheltens scale on T2 fluid-attenuated inversion recovery imaging, and global cognitive function was assessed using the Mini-Mental State Examination (MMSE) and the Montreal Cognitive Assessment scale (MoCA). At baseline, cognitive dysfunction was present in approximately 90% of the participants (MoCA < 26). Compared with the sham RIC group, RIC significantly decreased WMHs and improved cognitive function at 180 and 300 days, respectively. Using the same protocol, another randomized controlled trial was performed in 30 patients with cognitive impairment associated with cerebral small vascular disease (CSVD) (Wang Y. et al., 2017). After 1 year of treatment, there was a significant reduction in the volume of WMHs and a better performance in visuospatial and executive function in the RIC group. This may be partially modified by a reduction in plasma lipid levels (such as low-density lipoprotein), along with pulsation indices of the middle cerebral arteries. Moreover, in patients with subcortical ischemic vascular disease, compared with sham RIC, chronic RIC (5 min × 5 min, twice daily for 6 months) significantly improved visuospatial function (Liao et al., 2019). Although no significant difference was found between the two groups, a more pronounced reduction in WMH volume was observed in the RIC group (pre-treatment 84.00 vs. post-treatment 69.87, *P* = 0.060). In contrast, one small pilot randomized clinical study on 17 patients with CSVD reported that RIC failed to improve global cognitive function at a 1-year follow-up, although it significantly reduced the WMH volume (Mi et al., 2016). A possible explanation for this result is that the cognitive function of most individuals in both groups was not impaired at baseline (with a median MMSE score of 30 and MoCA score of 26 in the RIC group, and 28.5 and 27.5 in the sham group, respectively). This may cause a “ceiling effect” for the RIC. It is noteworthy that subjects in the sham group showed a tendency of cognitive decline, while it was preserved in the RIC group, indicating that RIC may slow down cognitive decline. However, further clinical studies are needed to confirm this finding. Damage of oligodendrocytes is a major pathological change in WM lesions (Alber et al., 2019), which is later discussed in detail (see section “Oligodendrocyte”).

Taken together, chronic RIC confers a protective effect toward VCI by reducing WM damage. It can improve cognitive function in patients who have been diagnosed with VCI and may prevent cognitive decline in patients with cerebrovascular diseases.

#### RIC Protects the Neurovascular Unit

##### Neurons

Neurons play a central role in the NVU. Neural dysfunction or death, especially in the hippocampus and prefrontal lobe, which are engaged in the learning and memory processes, is considered as a major cause of VCI (Deramecourt et al., 2012; Bartsch and Wulff, 2015; Meftahi et al., 2021). It has been shown that RIC is capable of reducing neural death and improve cognitive function in VCI animal models (Khan et al., 2015, 2018; Ren et al., 2018a). In a tMCAO rat model, for instance, a substantial loss of cholinergic neurons in the hippocampal CA1 region was observed 3 days after surgery, while RIC with three cycles of ischemia and reperfusion for 5 min each significantly protected against cell death and preserved cognitive function, as evaluated using the Morris water maze test (Hu et al., 2013).

Numerous mechanisms underly the protective effect of RIC on neurons, and the suppression of apoptosis might be one of them. Neuronal apoptosis is a crucial pathological change in cognitive function associated with acute or chronic cerebral hypoperfusion (Poh et al., 2020; Wan et al., 2020), which is characterized by downregulation of anti-apoptotic Bcl-2, upregulation of pro-apoptotic Bax, and activation of caspases (Wang X. X. et al., 2020). Thereby, anti-apoptosis is considered a target for VCI. RIC has been shown to preserve the cognitive function of rats after BCAO, and the upregulation of Bcl-2 in the hippocampus may at least partially contribute to this protective effect (Xu et al., 2011). The underlying mechanism is likely to be related to the activation of the JAK2–STAT3 pathway and the PI3K–pAkt pathway by RIC (You et al., 2019).

It appears that regulation of the autophagic process may also be involved in the protective effect of RIC. Autophagy is a lysosome-dependent cellular catabolic pathway, which enables cells to degrade damaged organelles and aggregated proteins for cellular and energy homeostasis (Wang X. X. et al., 2020). Accumulating evidence has indicated that autophagy is widely activated upon ischemic insult (Jiang et al., 2017; Deng et al., 2020). However, the role of autophagy in VCI is controversial. Several studies have indicated a protective role of autophagy (Yang C. et al., 2017; Wang F. et al., 2019; Wang N. et al., 2019; Thammisetty et al., 2021). In contrast, multiple studies have reported a detrimental role of autophagy in VCI, which aggregated neuronal death (Jia et al., 2015; Hu et al., 2017; Qin et al., 2018; Su et al., 2018; Xia et al., 2019; Zhang et al., 2019; Tian et al., 2020; Yang et al., 2020). Furthermore, others have indicated that impaired autophagy flux, characterized by elevated autophagosome formation and/or suppressed autophagy degradation, is tightly linked to cell death and cognitive impairment (Xu et al., 2020; Chen et al., 2021). RIC has been shown to alleviate neuronal death in the hippocampus and preserve WM integrity through the promotion of autophagy. In a BACS rat model, RIC for 2 weeks significantly upregulated the expression of Beclin1, LC3, Atg5-Atg12, and Atg 7, while downregulating the expression of p62 in the hippocampus and WM (Wang H. et al., 2017). The former proteins are important in the initiation and formation of the autophagosome and the latter, p62, can bind to the ubiquitined protein and LC3, serving as a marker of autophagic degradation, and decreased p62 levels are generally related to autophagy activation (Klionsky et al., 2016).

Taken together, RIC decreases neuronal death and preserves cognitive function at least partially via the suppression of apoptosis and promotion of autophagy.

##### Endothelial cells

It has been well established that RIC preserves endothelial function in healthy individuals (Jones et al., 2015; Rytter et al., 2020). Recently, the protective effect of RIC on endothelial function has also been proved in subjects with cerebrovascular diseases (Hyngstrom et al., 2020). This trial was conducted on 24 chronic stroke survivors (>6 months post-stroke), and RIC was performed on the unilateral thigh of the participants with five cycles of ischemia and reperfusion intervals for 5 min per cycle every other day for 2 weeks. Endothelial function, measured by brachial artery flow-mediated dilation (FMD), was significantly elevated in the RIC group after the treatment; however, no significant changes were observed in the sham RIC group. Although cerebral endothelial function was not evaluated in this study, FMD may be a reliable proxy for its determination as a marker of peripheral endothelial function (Pretnar-Oblak et al., 2006). Thus, it was postulated that RIC may improve cerebral endothelial function in stroke survivors, which is beneficial for cognitive performance.

The mechanism underlying the effect of RIC on endothelial function remains unknown. Nonetheless, previous studies in healthy subjects have addressed the participation of glucagon-like peptide (GLP)-1 receptor (Verouhis et al., 2019). In this study, twelve participants were enrolled, and endothelial dysfunction was induced by forearm ischemia for 20 min. The RIC consisted of four cycles of cuff inflation and deflation for 5 min per cycle was applied to the left thigh, which showed significant protection against endothelial dysfunction following I/R injury. This beneficial effect of RIC was abolished when the GLP-1 receptor antagonist exendin (9–39) was administered to the participants. Of note, the plasma concentration of GLP-1 did not change after RIC. A possible explanation is that GLP-1 may act locally, without being released into the circulation and relaying the protective effect of RIC by activating other pathways.

In conclusion, RIC can improve the endothelial function in healthy individuals as well as in chronic stroke survivors, which may be mediated by the activation of GLP-1 receptor.

##### Oligodendrocytes

Oligodendrocytes are extensively distributed in the WM and are important for axonal myelination. In rats subjected to BCCAO, RIC for 4 weeks significantly inhibited oligodendrocyte loss, promoted myelination in the corpus callosum, and preserved cognitive function compared to rats in the control group (Li et al., 2017). The underlying mechanism may involve the downregulation of Phosphatase and tensin homolog (PTEN) and upregulation of the phosphatidylinositol-3-kinase (PI3K)/Akt and the mammalian target of rapamycin (mTOR) signaling pathway (Li et al., 2017). The serine/threonine kinase Akt is involved in a variety of biological processes such as cell survival, growth, proliferation, and differentiation (Manning and Toker, 2017). A key kinase downstream of Akt is the mTOR, which gives rise to two distinct complexes: mTOR complex 1 (mTORC1) and complex 2 (mTORC2) (Saxton and Sabatini, 2017; Figlia et al., 2018). It has been reported that RIC upregulate the expression of mTOR and improved the cognitive function in a global brain ischemia mice model (Zare Mehrjerdi et al., 2013). The hyperactivation of the PI3K/Akt/mTOR pathway in transgenic mice leads to hypermyelination (Narayanan et al., 2009; Goebbels et al., 2010), while ablation of mTOR results in a deficit in oligodendrocyte differentiation and myelin loss (Ishii et al., 2019). PTEN is a negative regulator of the PI3K/Akt/mTOR signaling pathway through dephosphorylating PI3K to phosphatidylinositol 4,5-bisphosphate (Yehia et al., 2019). Thus, downregulation of PTEN may active the PI3K/Akt/mTOR signaling pathway, which in turn promotes myelination, reduces WM damage and improved cognitive function.

#### RIC Attenuates Oxidative Stress

There are evidence suggest that RIC is a viable antioxidant therapy for VCI. In a rat model of global cerebral ischemia (GCI) induced by the occlusion of the bilateral carotid arteries for 20 min followed by reperfusion, the level of ROS were elevated, while the level of antioxidants were consumed in the brain (Ramagiri and Taliyan, 2017b), indicating a state of oxidative stress. And this was accompanied by neuronal damage in the hippocampus and cognitive dysfunction. RIC (three cycles of 10 min ischemia and reperfusion) applied immediately following reperfusion significantly reduced ROS, such as MDA, and prevented the reduction of the defensive enzyme glutathione (GSH).

By using the same animal model, others have also observed the antioxidant properties of RIC by elevating heme oxygenase-1(HO-1) (Ramagiri and Taliyan, 2017a). HO-1 is an important antioxidant that can degrade pro-oxidant heme to cytoprotective carbon monoxide, iron, and biliverdin (Bauer and Raupach, 2019). Following reperfusion, RIC significantly improved cognitive function, increased the expression of HO-1 and GSH, decreased the expression of MDA, and reduced neuroinflammation and CA1 neuronal death. In the presence of the HO-1 inhibitor, tin protoporphyrin IX (SnPP), the protective effect of RIC against I/R injury-induced cognitive decline was abolished, suggesting that HO-1 plays an important role in RIC-induced neuroprotection.

One mechanism of RIC protection against oxidative stress is the activation of Nuclear factor-like 2(Nrf2). Nrf2 is a key regulator of the antioxidative stress defense system that maintains the equilibrium of redox responses. Under pathological conditions, Nrf2 dissociates from Keap-1, translocates to the nucleus, and binds to the antioxidant response element, which regulates the expression of many antioxidant genes (Liu et al., 2019). Nrf2-knockout mice are more susceptible to cerebral I/R injury (Yang et al., 2019) and exhibit exacerbated WM damage under chronic hypoperfusion (Sigfridsson et al., 2020). Upregulation of Nrf2 signaling through pharmacological or genetic approaches protects against WM injury, neuronal death, and cognitive impairment in VCI models (Sigfridsson et al., 2018; Mao et al., 2019). In a GCI mouse model, RIC was performed as four cycles of ischemia and reperfusion for 5 min per cycle before surgery (He et al., 2019). Compared with the sham group, animals in the experimental group showed a significant restoration in cognitive function, assessed using the Morris Water Maze, 7 days after ischemia, along with an upregulation of Nrf2 and GR in the brain. This beneficial effect of RIC may be attributed to an increase in endothelin-1 (ET-1) in the plasma, which may act as an upstream mediator of hydrogen sulfide in the brain, and in turn upregulate Nrf2 expression.

In summary, the above findings indicate that RIC is able to attenuate the oxidative stress by decreasing the ROS expression and improving the antioxidant system. The activation of Nrf2 may play an important role in this process, which induces the expression of antioxidant enzymes.

#### RIC Mitigates Inflammation Responses

Numerical studies have reported the effect of RIC in ameliorating systemic inflammation and neuroinflammation. In a randomized controlled prospective study enrolled 58 patients with sICAS aged 80–95 years, repeated RIC for 30 days safely reduced inflammation biomarkers (Meng et al., 2015). In this trial, 30 patients in the RIC group received 5 cycles of ischemia and reperfusion for 5 min per cycle, twice daily on bilateral arms, and 28 patients in the control group received sham RIC (inflation to a pressure of 30 mmHg). All patients received standard medical care. Although cognitive function was not assessed, the levels of plasma hsCRP, IL-6, leukocyte count, and platelet aggregation rates were significantly decreased in the RIC group compared to those in the control group, suggesting that the underlying mechanisms of RIC involve the relief of systemic inflammatory response.

Another clinical study examined the efficacy of RIC on post-stroke cognitive impairment in patients with first-time non-cardiac ischemic stroke (Feng et al., 2019). During the acute phase (<14 days after onset), 104 patients were enrolled and randomized into control or RIC groups. Both groups received standard treatment. RIC were performed with 5 cycles of unilateral arm ischemia and reperfusion for 5 min per cycle for 6 months. Participants in the RIC group showed a lower ICAM-1 level and a higher MoCA scores compared with the control group, indicating a better global cognitive function. Specifically, participants in the RIC group performed better on the cognitive domains of visuospatial and executive functioning and attention.

In consistent with the clinical studies, in a VCI mouse model, 2 weeks of RIC with four cycles of ischemia and reperfusion for 5 min per cycle significantly downregulated the expression of ICAM-1, VCAM-1, glial fibrillary acidic protein, and ionized calcium binding adaptor molecule-1 (IBA-1) in the brain parenchyma, accompanied by improved cognitive function, blood flow, and decreased WM damage and amyloid-β (Aβ) aggregation (Khan et al., 2015). ICAM-1 and VCAM-1, derived from ECs, are important adhesion molecules that regulate leukocytes crossing the BBB (Haarmann et al., 2015; Muller, 2019). Opening of the BBB initiates local inflammation and compromises cerebral vascular function, which in turn may lead to impairment of Aβ clearance, WM integrity, and cognitive function. Thus, downregulation of these adhesion molecules may mitigate neuroinflammation and help maintain cognitive function.

Overall, RIC can attenuate the systemic inflammation and neuroinflammation response, including reduced hsCRP, IL-6, leukocyte counts, adherence molecules, reactive microglial and reactive astrocytes. It is therefore important in maintaining the brain health and normal cognitive function.

## Discussion

Over the past decades, substantial progress has been made in clarifying the pathobiologies of VCI. However, no pharmaceutical therapies targeting these mechanisms have been applied clinically. RIC is a potential intervention to prevent the decline of cognitive function and even reverse cognitive impairment in patients with VCI, particularly visuospatial ability. It can be safely applied in daily life as an adjuvant treatment for alleviating risk factors, such as recurrent stroke and prehypertension. Meanwhile, RIC can act on multiple pathobiological changes in VCI through various signaling pathways to exert neuroprotective effects. This might render it even more efficient for VCI than single target drugs.

However, RIC research on VCI is still in its early stages. The efficacy of RIC on VCI has mainly been proven in small-scale clinical trials, which makes it difficult to avoid selective bias. Thus, large-scale trials are needed to confirm these findings. WMHs have been frequently used as biomarkers for the assessment of treatment efficacy. It was reported that fewer patients were needed if using visually rated WM lesion progression, instead of cognitive scoring scales, in clinical trials of CSVD (Schmidt et al., 2012). This may be important for proof-of-concept studies.

In animal studies, BCCAO and BCAS are the most commonly used methods to induce typical pathogenic changes in VCI. However, it can only mimic VCI of large vessel origin. Using animal models of small vessel diseases, such as SHR, may help to fully evaluate the effectiveness and to elucidate the mechanisms of RIC (Hainsworth et al., 2017). In addition, most animal studies have focused on the protective effect of RIC within the brain and did not account for the initiation or signal transduction of RIC in the periphery, although this has been extensively described in other animal models. It seems that the eNOS/NO/nitrite system at least partially mediates the protective effect of RIC from triggered organs to the brain. Considering the important role of ECs in this process, endothelial function, as measured by FMD, may be a simple, straightforward, and efficient parameter to evaluate RIC response in clinical settings; however, this hypothesis remains to be verified.

From a methodological viewpoint, the optimal algorithm for RIC on VCI has not been scientifically proven, while most of the clinical studies in this field used a protocol of 5 min × 5 min cycles; however, 3 min × 10 min was commonly used in animal studies. It has been reported that the effect of RIC on cardioprotection presented a U-type tendency in a mouse model (Johnsen et al., 2016). Four to six cycles of RIC provided significant protection, with no further gain after eight cycles. Two minutes and 5 min of ischemia interval offered similar protection, while 10 min abrogated this effect. However, ischemia intervals lasting for 10 min provided cerebral protection in the VCI animal model. It seems that the optimal protocol for the brain is different from that for the heart; this should be explored in future research.

Despite these limitations and challenges, RIC has therapeutic advantages owing to its multitarget characteristics, which could provide a synergistic effect in VCI. Importantly, the effect of RIC on each pathobiology of VCI has been confirmed in both animal and human studies. Therefore, RIC appears to be a promising intervention to prevent the occurrence and development of VCI.

## Conclusion

To date, there is no effective pharmacotherapy for VCI to slow down or even reverse cognitive decline, especially in patients with mild VCI. Cerebrovascular risk factors, such as stroke and hypertension, play a detrimental role in VCI, which results in large or small vessel injuries and various pathological changes within the brain. RIC can act as a multitarget therapy to combat VCI by reducing recurrent stroke, lowering high blood pressure, enhancing CBF, alleviating WM damage, protecting components with in the NVU and attenuating oxidative stress and inflammatory response. However, this non-invasive and cost-effective intervention warrants further larger clinical studies and in-depth animal studies to support its beneficial effects.

## Author Contributions

RX and Z-NG conceived the perspective of the work. RX searched the literature and drafted the manuscript. QH and YW critically revised the article. YY and Z-NG was responsible for checking the whole manuscript. All authors contributed to and approved to the final manuscript.

## Conflict of Interest

The authors declare that the research was conducted in the absence of any commercial or financial relationships that could be construed as a potential conflict of interest.

## Publisher’s Note

All claims expressed in this article are solely those of the authors and do not necessarily represent those of their affiliated organizations, or those of the publisher, the editors and the reviewers. Any product that may be evaluated in this article, or claim that may be made by its manufacturer, is not guaranteed or endorsed by the publisher.
